# Self-medication with nutritional supplements and herbal over-thecounter products

**DOI:** 10.1007/s13659-011-0029-1

**Published:** 2011-11-12

**Authors:** Tolga Eichhorn, Henry Johannes Greten, Thomas Efferth

**Affiliations:** 1 Department of Pharmaceutical Biology, Institute of Pharmacy and Biochemistry, Johannes Gutenberg University, Staudinger Weg 5, 55128 Mainz, Germany; 2 Heidelberg School of Chinese Medicine, Karlsruher Straße 12, 69126 Heidelberg, Germany; 3 Biomedical Sciences Institute Abel Salazar, University of Porto, Porto, Portugal

**Keywords:** botanicals, contamination, complementary and alternative medicine, drug interactions, geriatric, gynecology, insomnia, hepatotoxicity, menopause, nephrotoxicity, over-the-counter, pain, phase I/II enzymes, pharmacognosy, pharmacovigilance, phytochemicals, phytotherapy, phytovigilance, quality control

## Abstract

In recent years, the popularity increased for nutritional supplements and herbal products. Prescription drugs, but not herbal therapies are paid by health insurances. They are sold over-the-counter (OTC) on the patients’ own expense. However, there are potential risks of self-medication, *e.g.* incorrect self-diagnosis, severe adverse reactions, dangerous drug interactions, risk of addiction etc. They are often used by patients at their own discretion without knowledge of and control by their physicians. Certain users are at risk of intoxication. Multiple medications taken by older patients increase the risk for adverse drug reactions, drug-drug interactions, and compliance problems for this age group (polypharmacy). Herbals should be discontinued prior to operations to avoid interactions with anesthetics or anticoagulants. Herbal preparations may also be carcinogenic or interfere with cancer treatments. Pregnant women use various OTC preparations. However, in many cases, it is unclear whether their use is safe for mother or baby. Self-medication with herbals is also largely distributed among anxious and depressive patients, and patients with other conditions and symptoms. The popularity of herbal products has also brought concerns on quality, efficacy and safety. Cases of botanical misidentification, contaminations with heavy metals, pesticides, radioactivity, organic solvents, microbials as well as adulteration with chemical drugs necessitate the establishment of international quality control standards. Hepatotoxic effects have been reported for more than 300 plant species, and some commonly used herbs have been demonstrated to interact with Western medication. Health care professionals have a critical responsibility assessing the self-care ability of their patients. Databases are available for pharmacists with information on action, side effects and toxicities as well as herb-drug interactions. There is a need for established guidelines regarding the correct use of nutritional supplements and herbal OTC preparations (phytovigilance). Physicians, pharmacists, and other health care professionals have to counsel patients and the general public on the benefits and risks associated with herbal drugs. Information centers for consumers and general practitioners are needed, and convincing evidence on safety and efficacy of herbal products has to be demonstrated in placebo-controlled, double blind and randomized clinical trials.

### Introduction

According to estimations of the World Health Organisation, 70-80% of the world populations rely on non-conventional medicine mainly of herbal sources in their primary health care.[1,2] While patients in developing countries depend on herbal medicine because they cannot afford the costs for prescription Western medicine, the situation is *vice versa* in industrialized countries. In recent years, the popularity increased for over-the-counter (OTC) health foods, nutraceuticals, and medicinal products from plants or other natural sources in industrialized countries. Prescription drugs but not herbal therapies are paid by the health insurance system. They are sold over-the-counter on the own expense of the patients. Therefore, herbal OTC products and complementary and alternative medicine (CAM) attracts patients from higher social classes. This also indirectly indicates that the public might not be satisfied with their Western medical treatment.[3]

If herbal medicines meet rigourous quality control in production and evidence-based standards in safety and efficacy, they would have the potential to reduce costs for the health insurance systems. The frequent reluctance of the pharmaceutical industry is understandable in terms of the difficult patent situation with phytotherapy and the

*To whom correspondence should be addressed. E-mail: efferth@uni-mainz.de possibilities to earn money with herbal products and nutritional supplements. However, faced with ever-increasing costs for medications, phytotherapy bears a considerable potential for future health care in times of bursting in health systems. As compared to conventional patients, CAM patients cause lower costs.[4]

Self-medication is defined as the use of medicines to treat self-recognized conditions or symptoms. One benefit of self-medication may be the active role of the patient in his/her own health care. However, there are potential risks of self-medication, *e.g.* incorrect self-diagnosis, delays in seeking medical advice when needed, severe adverse reactions, dangerous drug interactions, masking of a severe disease, risk of addiction, and abuse.[5]

Herbs are classified as dietary supplements (not drugs), and manufacturers are therefore not required to provide proof of efficacy or safety before selling these substances. The general community perception that herbal preparations are safe may lead to inappropriate use. Improper intake may causer unwanted side effects and toxicities both in industrialized and developing countries.[6,7]

CAM is actually a vast collection of disparate, unrelated therapies and products, ranging from treatment modalities that effectively enhance quality of life to promising herbal remedies.[8] Complementary therapies such as music and massage, herbal teas, yoga, tai chi, meditation, and many other techniques that augment Western medical treatment. A lack of regulations leaves consumers at the mercy of those who promote unproved remedies. Herb-drug interactions frequently require that patients stop taking herbal remedies prior to surgery (to prevent interactions with anesthetics and anticoagulant effects), radiation (due to potential for increased radiosensitivity of healthy tissues), and chemotherapy (to prevent herb-drug interactions).

Although laws pertaining to dietary supplement labeling prohibit specific claims for the treatment or prevention of disease, these products are widely used as CAM therapies. Complex issues persist, which have to be addressed when consulting patients.[9]

Dietary supplements are often used by patients at their own discretion without knowledge of and control by their physicians. Patients may anticipate physicians' disapproval of their use, or not realize that it is important for the physician to know what they are taking. Therefore, it is imperative that patients are asked unbiassed questions about current and past use of herbals and alternative therapies. Even when physicians are aware of such use, they feel poorly trained to identify the constituents and effects.[10]

There is still limited evidence to evaluate the efficacy of the approximately 20,000 available herbal products. Because herbs contain potent bioactive substances and are often marketed to treat specific diseases, many have argued that they should be subject to more stringent regulations.[11] To improve the safety and consistency of herbs, additional research is needed to define the pharmacology, stability, and bioavailability of these products. Approaches for regulation and common-sense guidelines are necessary.

### 1 Target Groups of Self-Medication

In principle, self-medication can be applied by the general public. However, certain users of herbs are at high risk of intoxication.[12] These include chronic users consuming large amounts or a great variety, malnourished or undernourished persons and patients on long term medication. Certain plant toxins may be gender-selective in their action. Here, we focus on groups of patients, which frequently take nutritional supplements and herbal OTC products to treat their ailments, conditions and symptoms and which might be specifically at risk.

#### Elderly

Since older adults are a growing sector of the population in many countries, it is important to know the prevalence of their herbal product use. To estimate the prevalence of self-medication in the elderly, Vacas Rodilla et al.[13] performed a cross-sectional descriptive study. A total of 240 patients 75 years of age or older have been assessed in an urban primary health care centre in Spain. Self-medication frequency was 31%, with 23% being pharmacological and 15% herbal. The drugs most used in self-medication were analgesics and cold remedies. The pharmacy was the most usual source (49%). Accumulating drugs in homes was a very extensive practice.

The plethora of medications taken by older patients increases the risk for adverse drug reactions, drug-drug interactions, and non-compliance for this age group. This situation has been termed polypharmacy, which is defined as the use of (too) many different medications, which increases the probability of side effects and drug interactions. As pointed out by Tam-McDevitt,[14] polypharmacy can be an issue in any age group, but seems to be especially a problem for the elderly, who consume more medications than any other patient group. Factors such as the presence of multiple co-morbid conditions, advances in pharmacotherapy, and increased availability of OTC and herbal supplements for self-treatment can all contribute to polypharmacy in this population. Physiologic changes associated with aging may alter the pharmacokinetic and pharmacodynamics of drug metabolism, which in turn affects potential drug toxicities.

It is important to avoid health complications due to the concomitant use of diverse medications and herbs. González-Stuart[15] reported that elderly are especially vulnerable, since the human body's physiological activities, such as renal and hepatic detoxification and clearance usually decrease with age. Elderly taking combinations of various medications and herbal supplements are also more at risk to experience herb-drug interactions.

Musculoskeletal pain in the elderly is common and disabling. As the conditions causing rheumatic pain, including osteoarthritis, inflammatory arthritis and certain soft-tissue conditions are mostly not curable, pain control is paramount to maintain quality of life. Pain management is multimodal and tailored to the individual patient. Although herbal products and nutritional supplements are commonly used by patients, studies of their efficacy and safety, especially in the elderly, are limited.[16] The use of any pharmacological agent in the elderly - being it standard pharmacology or herbal remedy - should be tempered with caution regarding increased sensitivity to medications, herbal-drug interactions and associated co-morbidities. Therefore, the elderly will often require down-adjustment of dosage and careful attention to the risk/benefit ratio of the treatment. However, barriers to effective pain management from the perspective of both the patient and the healthcare professional still exist, and have to be overcome by educational efforts.

Results from clinical trials demonstrate that standardized leaf extracts of *Ginkgo biloba* reduce the symptoms of age-associated memory impairment and dementia, including Alzheimer's disease, and may be of benefit in treating intermittent claudication.[17] In addition, *Ginkgo biloba* extract may be useful in preventing and treating cardiovascular disease (CVD), particularly ischemic cardiac syndrome.[18] Several herbal or alternative therapeutics can significantly elevate blood pressure or cause interactions with cardiovascular drugs. Vora and Mansoor[10] reviewed the epidemiology of alternative therapy use, and selected several important herbal or other supplements that patients with hypertension and cardiovascular diseases may be taking.

#### Surgical Patients

The American Society of Anesthesiologists (ASA) recommends that all herbal medications should be discontinued two to three weeks prior to an elective surgical procedure.[19] King and colleagues[20] performed a retrospective review of surgery patients presenting to the Anesthesia Preoperative Evaluation Clinic (APEC) at the University of Kansas Hospital (Kansas City, KS, U.S.A.). Approximately one-fourth of patients indicated the use of natural products. Patients taking natural products were significantly older, were more likely to undergo cardiac or chest surgery, and were more likely to take more prescription and non-prescription medications.

At the University of Colorado Health Sciences Center (Denver, Colorado, USA) Norred et al.[21] surveyed 500 elective surgical outpatients about alternative medicines taken during the two weeks prior to surgery. Of 500 patients surveyed, 51% preoperatively took herbs, vitamins, dietary supplements, or homeopathic medicines. Two or more categories of alternative medicines (herbs, vitamins, dietary supplements, or homeopathic medicines) were consumed by 24% of patients. Twenty-four percent of surveyed patients consumed 50 different herbs, 41% took 9 types of vitamins, 44% took 31 types of dietary supplements, and 1% of patients took homeopathic *Arnica*. Classification by potential adverse effects revealed that 27% of surgical patients consumed alternative medicines that may inhibit coagulation, affect blood pressure, cause sedation, have cardiac effects, or alter electrolytes.

Rowe and Baker[22] reviewed research on the possible benefits and risks of commonly used herbal medications such as *Arnica montana*, St. John's wort, bromelain, *Echinacea, Ginkgo biloba, Ephedra*, valerian, and others, focusing on their potential impact during the perioperative period of aesthetic surgery. A large percentage of patients undergoing plastic surgery use one or more herbal medications, but the disclosure of such medications to allopathic providers is often incomplete. In addition, patients may not understand the importance of discontinuing such medications before surgery.

Dental professionals routinely treat patients taking prescription, nonprescription, and herbal medications that are known or have the potential to alter bleeding. Herbal supplements are widely used for a variety of indications, and both patients and health care practitioners are often unaware of the potential consequences. Decision-making strategies, such as interpretation of laboratory tests etc. should be applied. Herbal supplements must be discontinued two weeks prior to receiving invasive dental surgical procedures. Dental practitioners should implement risk reduction strategies to minimize adverse bleeding complications associated with dental treatment.[23]

#### Patients with Tumors or Benign Hyperplasias

Herbs and natural products may also be carcinogenic or interfere with cancer treatments. Some herbs may have estrogenic effects and compete with hormone cancer therapies, whereas others interfere with chemotherapy treatment or may induce recurrence of cancer.[24] Between 7% and 48% of cancer patients take herbal medicines after diagnosis.[25] Despite known interactions with conventional cancer treatments and contraindications for some herbal remedies with specific cancers, reliable information resources for patients are very limited. Identifying cancer patients' information needs and preferences is the first step in creating a suitable resource for both the public and professionals advising them.

PC-SPES is a potent eight-herb formulation sold directly to consumers. It has promising efficacy in the treatment of prostate cancer. The product induces a castrate status, resulting in a 50% or greater prostate-specific antigen reduction in the majority of men with androgen-dependent prostate carcinoma and in more than one half of the men with androgen-independent prostate cancer.[26] The duration of response is not yet clear. The efficacy of PC-SPES appears to exceed that of androgen ablation alone, but is not necessarily separate from an estrogenic effect. Common side effects include gynecomastia, nipple tenderness, loss of libido, and impotency. The mechanisms of action may involve down-regulation of the androgen receptor, induction of apoptosis and increased expression of p53. Two bioactive compounds in PC-SPES are baicalin and oridonin. FDA has warned consumers to stop taking the dietary supplement/herbal product PC-SPES because they contain undeclared prescription drug ingredients that could cause serious health effects if not taken under medical supervision. In February 2002, the manufacturer of the product in U.S.A. voluntarily recalled PC-SPES nationwide.[27]

Phytotherapeutic agents have also gained widespread use in the treatment of benign prostatic hyperplasia and lower urinary tract symptoms. Although numerous mechanisms of action have been postulated, it is still uncertain which are responsible for clinical response. The efficacy of these agents has not been conclusively proven.[28]

#### Pregnant Women

Pregnant women use a range of OTC preparations, including analgesics, antihistamines, antacids and a variety of herbal preparations.[29] However, in many cases, it is unclear whether their use is actually safe in the short- or long-term for mother or baby. Few studies about effects of herbs have been conducted in the general population, and fewer still have been published about certain risk groups. Because the perinatal nurse has two patients to consider when caring for a pregnant woman, he or she has two equally important mandates: to help the mother without harming the fetus.[30]

Tiran[31] and Holst et al.[32] reported on safety and efficacy of the most commonly used herbs to enable midwives to give evidence-based information to pregnant women. A total of 578 expectant mothers at least 20-weeks pregnant were surveyed. Fifty-eight percent of the participants used one or more herbal remedies. The most commonly used herbal preparations during pregnancy were ginger, cranberry, raspberry leaf, chamomile, peppermint and *Echinacea*. Although 14 studies focusing on the safety and/or efficacy of these herbals in human pregnancy were identified, the authors felt that there is limited documentation on the safety and efficacy of many herbs commonly used during pregnancy.

Dugoua et al.[33] focused on the use, safety and pharmacology of blue cohosh during pregnancy and lactation in U.S.A.. Approximately 64% of midwives use blue cohosh as a labour-inducing aid. Blue cohosh taken at the time of delivery may cause (1) perinatal stroke, (2) acute myocardial infarction, profound congestive heart failure and shock (3) severe multi-organ hypoxic injury, and (4) in rare cases blue cohosh possesses abortifacient properties. There is *in vitro* evidence that blue cohosh may have teratogenic, embryotoxic and oxytoxic effects. In lactation, the safety of blue cohosh is unknown. Based on the available scientific information, blue cohosh should (1) be used with extreme caution during pregnancy, (2) be used only under medical professional supervision, and (3) not be available to the public as an OTC product. There is an urgent need to conduct retrospective or prospective cohort studies of midwifes using blue cohosh to determine its safety.

**Slimming Persons**

OTC dietary supplements to treat obesity appeal many patients for weight loss. Non-prescription products available to control weight include herbal dietary supplements.[34,35] The introduction of orlistat as OTC product represents the only Food and Drug Administration (FDA)-approved product for weight loss currently in that category, since phenylpropanol-amine (PPA) was withdrawn by FDA.

Although evidence of modest weight loss after *Ephedra*-caffeine ingestion exists, potentially serious adverse effects have led FDA to ban the sale of these products. Guar gum and chitosan appear to be ineffective. Because of insufficient or conflicting evidence regarding the efficacy of conjugated linoleic acid, ginseng, glucomannan, green tea, hydroxycitric acid, L-carnitine, psyllium, and St. John's wort in weight loss, physicians should caution patients about the use of these supplements.[36]

**Dismenorrhea Patients**

Conventional treatment for primary dysmenorrhoea (painful menstruation) has a failure rate of 20% to 25%. Chinese herbal medicine may be a suitable alternative treatment. Zhu et al.[37] selected and evaluated 39 randomised controlled trials with a total of 3475 women involving Chinese herbal medicine *versus* placebo, no treatment, conventional therapy, heat compression, acupuncture or massage. Chinese herbal medicine resulted in significant improvements in pain relief, overall symptoms when compared to standard drugs. Chinese herbal medicine also resulted in better pain relief than acupuncture and heat compression. Although the review found promising evidence supporting the use of Chinese herbal medicine for primary dysmenorrhoea, the results were limited by the poor methodological quality of the included trials.

**Menopausal Women**

Postmenopausal women frequently use OTC therapies for the treatment of hot flashes. Herbal remedies include black cohosh, isoflavones, red clover, soy, vitamin E, ginseng, evening primrose oil, wild yam, kava, melatonin and others. A recent review on clinical evidence for OTC products for relief of hot flashes in menopausal women showed that the published literature is conflicting regarding efficacy.[38]

Haimov-Kochman et al.[39] examined the long-term safety and herb-drug interactions of commonly used herbal therapy such as soy, black cohosh, ginseng and vitamin E reported in the literature. The authors found that even carefully designed studies on herbal treatments for vasomotor menopausal symptoms did not specifically address safety issues.

**Anxious and Depressive Patients**

Anxiety disorders are among the most common psychiatric disorders that affect all age groups of the general population. The preferred treatment is with antidepressant or anti-anxiety drugs that have adverse effects, such as sedation, impaired cognition, ataxia, aggression, sexual dysfunction, tolerance and dependence. Several studies have suggested that self-medication with herbals is largely distributed among anxious and depressive patients.[40] Herbal remedies, including kava (*Piper methysticum*), are effective treatments, at least in mild to moderate cases of anxiety.[41] Kava is a social and ceremonial herb from the South Pacific. It is available in the west as OTC preparation. The biologically active kavalactones reveal sedative, anxiolytic, antistress, analgesic, local anaesthetic, anticonvulsant and neuroprotective properties. The pharmacological properties of kava are postulated to include blockade of voltage-gated sodium ion channels, enhanced ligand binding to gamma-aminobutyric acid (GABA) type A receptors, diminished excitatory neurotransmitter release due to calcium ion channel blockade, reduced neuronal reuptake of noradrenaline (norepinephrine), reversible inhibition of monoamine oxidase B and suppression of the synthesis of eicosanoid thromboxane A(2), which antagonises GABA(A) receptor function. Kava and kavalactones are effective in the treatment of anxiety due to various medical conditions.[42] Until recently, the adverse effects attributed to kava use were considered as mild or negligible, except for the occurrence of skin lesions. Kava dermopathy occurs only with prolonged use of large amounts of kava and is reversible on reduced intake or cessation. Rare cases of interactions have occurred with pharmaceutical drugs that share mechanisms of action with kavalactones. Cases of severe liver toxicity associated with kava intake have been reported. However, a direct causal relationship with kava use has been difficult to establish in the majority of cases.

**Patients Suffering from Insomnia**

Insomnia is a common condition resulting in significant clinical and economic consequences.[43] Meolie et al.[44] evaluated the safety and efficacy of non-prescription therapies used for insomnia. Therapies include passion flower, valerian,

Jamaican dogwood, hops, California poppy, chamomile, lemon balm, St. John's wort, kava kava, wild lettuce, scullcap, *Patrinia* root, first-generation histamine-1-receptor antagonists, and others. Although randomized, placebo-controlled studies were available for some drugs, rigorous scientific data supporting a beneficial effect are not found for the majority of herbal supplements used for treating insomnia symptoms. There are significant potential risks associated with the use of Jamaican dogwood, kava kava, alcohol, and I-tryptophan.

#### Dermatological Patients

Herbal preparations and isolated phytochemicals are increasingly used for dermatological ailments.[45,46] In acne therapy, tea tree oil, *Mahonia*, and *Saccharomyces* may become standard treatments. *Mahonia, Hypericum, Glycyrrhiza* and some traditional Chinese medicines appear promising for atopic dermatitis. Some plant-derived substances such as dithranol and 8-methoxypsoralen (in combination with UV-A irradiation) are already accepted as standard treatments in psoriasis. *Mahonia* and *Capsicum* (capsaicin) are also promising candidates. Oral administration and topical application of antioxidant plant extracts (green and black tea, carotenoids, coffee, and many flavonoids from fruits and vegetables) can protect skin from UV-induced erythema, early aging, and irradiation-induced cancer. Hair loss and vitiligo are also traditional fields of application for herbal products.

#### Ulceration

Aphthous ulceration is a disease of the intra-oral mucosa that is often self-managed by OTC medication. However, there are no randomized controlled studies to demonstrate OTC preparations do more than manage symptoms.[47] Exceptions are OTC cyanoacrylate products and CankerMelts GX patches, which contain *Glycyrrhiza glabra* (licorice) extracts. CankerMelts alters the course of the condition by reducing lesion duration, size, and pain. It may be as effective as the prescribed drug, Amlexanox, in reducing pain and speeding healing.

#### Immunodeficient Patients

The use of herbs has been advocated as alternative treatment for human immunodeficiency virus-related illness. Kassler et al.[48] interviewed 114 randomly selected patients. Twenty-five participants (22%) reported using one or more herbal products in the past three months. Of those taking herbs, six (24%) were unable to identify the herb that they had used. Twelve patients (48%) reported taking herbs for longer than 90 days. Of those taking herbs, five (20%) stated that their primary medical provider was unaware of their herb use, and four (16%) were involved in clinical drug trials while using herbs. Adverse effects include dermatitis, nausea, vomiting, diarrhea, thrombocytopenia, coagulopathies, altered mental status, hepatotoxicity, and electrolyte disturbances.

#### Influenza Patients

Influenza infection is still a leading cause of morbidity and mortality, accounting for 20–25 million doctor visits and 36,000 deaths per year in the U.S.A. Conventional therapies for colds and flu focus primarily on temporary symptom relief and include OTC antipyretics, anti-inflammatories, and decongestants. Treatment for influenza also includes prescription antiviral agents and vaccines for prevention. Some herbal drugs (*Echinacea spp., Sambucus nigra*, larch arabinogalactan, *Astragalus membranaceous, Baptisia tinctoria, Allium sativa, Panax quinquefolium, Eleutherococcus senticosus, Andrographis paniculata*, olive leaf extract, and *Isatis tinctoria*) and nutritional supplements (vitamins A and C, zinc, high lactoferrin whey protein, N-acetylcysteine, and DHEA) may support prevention and treatment.[49]

#### Patients with Eating Disorder

The rhizome and roots of the South-American plant, Ipecacuanha (ipecac, *Cephaelis ipecacuanha* or *C. acuminata)* is used as emetic and expectorant. The use of gastric emptying techniques, including ipecac-induced emesis, in the management of poisoned patients has declined significantly in recent years. Ipecac syrup was administered to patients prior to referral to the emergency department in attempts to start the gastric emptying process as early as possible. Ipecac abuse occurs predominantly among adolescent and young adult females who are either experimenting with purging or have an eating disorder.[50] Studies to determine the effectiveness of ipecac syrup demonstrate that it induces vomiting in a high percentage of people and that it decreases the gastrointestinal absorption of ingested substances. However, the effectiveness of ipecac syrup in affecting patient outcome has not been studied in adequate clinical trials. Its effectiveness in preventing drug absorption has only been documented for a limited number of substances.[51]

Psychiatric co-morbidity is common. Death can occur and is usually of cardiac origin. Morbidity includes myocarditis with arrhythmias, myositis, gastroesophageal conditions, diarrhea, and metabolic abnormalities (alkalosis, hypokalemia, dehydration). The injuries are reversible upon cessation of ipecac use. Emetine is an active alkaloid in ipecac, which may account for these effects.

### 2 Risks Associated with Herbal Self-Medication

#### Missing Quality Control

The increase in popularity of herbal products has also brought concerns and fears on the professionalism of practitioners, and quality, efficacy and safety of their treatment methods and remedies available on the market.[52] Herbal toxicity and adverse effects are well documented in classical Chinese medical textbooks. During the past decade severe and sometimes even life-threatening intoxications after intake of OTC herbal products became aware to the public media. Toxicity associated with herbal OTC therapy can derive from[53]:

(1) Correctly identified medicinal plants with unknown toxicity.

(2) Incorrectly identified medicinal plants with toxic activity. Botanical misidentification or mislabeling of plant material can play a role for toxic reactions in humans.

(3) Contamination of herbs with pesticides, heavy metals, and radioactivity taken up from the soil as well as organic solvents and microbial contamination due to unprofessional processing of harvested herbs.

(4) Substitution or adulteration with other herb or chemical drugs may deliberately occur for economic reasons, if a faked herb is cheaper than the original replaced one or for increasing the activity of an herbal mixture by adding a drug with well-known activity for the same indication.

An important role to prevent toxicities associated with low quality herbal products plays the establishment of international quality control standards[54]:

- The correct identification of medicinal plants is of utmost importance, since wrongly identified plants may contain poisonous ingredients.
- Standardization of production of herbal prescriptions by international quality guidelines such as Good Sourcing Practice (GSP) to guarantee authentication of medicinal plants, Good Agricultural Practice (GAP), Good Laboratory Practice (GLP), Good Manufacturing Practice (GMP), and Good Clinical Trial Practice (GCTP).
- Processing procedures of herbal products have to be standardized.
- Chromatographic fingerprinting analysis can disclose the detectable ingredients composition and concentration distribution.
- Quality control has not only the task to ensure proper composition of herbal prescriptions but also to avoid contamination with mycotoxins, pesticides, heavy metals, or other chemical toxins. Furthermore, faked herbal prescriptions with adulteration of drugs from Western medicine, *e.g.*, with glucocorticoids, have to be banned.
- Strengthening research on the physiological and pharmacological activity of herbal remedies.

### Toxicity

Hepatotoxic effects have been reported for more than 300 plant species, including *Heliotropium, Crotalaria, Senecio* and *Symphytum*.[55–57] In Africa or Central America, intoxication is sometimes endemic, since these plants are often used for making tea. In Western countries, cases of herb-induced hepatitis have been observed after use of preparations containing *Symphytum* or Chinese herbs. The pyrrolizidine alkaloid-containing germander (*Teucrium chamaedrys)* was used for weight loss. *Atractylis gummifera* is used for medicinal purposes or for chewing gum, but is hepatotoxic. Fatal liver accidents may occur after ingesting *Callilepis laureola*, a herb used by the Zoulous in Natal for medicinal purposes. These examples emphasize the importance of remembering that herbal medicine is not harmless.

Ulbricht et al.[58] identified more than 80 herbs or herbal products (including plants, fungi, algae, and common constituents) showing clinical interactions with prescription and OTC drugs. Drugs with anticoagulant/antiplatelet activity (*e.g.* warfarin, aspirin) were frequently implicated in herb-drug interactions. Because many herbs have demonstrated adverse effects on the liver, the potential for interaction with hepatotoxic agents (such as acetaminophen) is also significant.[59]

Herbal medicines are metabolized by phase I/II enzymes, including cytochrome P450 enzymes (CYP) and glucuronosyltransferases (UGTs) and translocated by phase III transporters such as P-glycoprotein (Pgp), increasing the likelihood of hepatotoxicity.[60] Examples include include black cohosh, *Echinacea*, garlic, piperine, ginseng, ginger, *Gingko biloba*, green tea, kava, milk thistle, St. John's wort, and others.

It is common for individuals with renal insufficiency to supplement their prescriptive treatment with non-prescription medications, herbal medicines, nutritional supplements and vitamins. Renal insufficiency may put individuals at an even higher risk for adverse events.[61,62]

### Herb-Drug Interactions

Based on available clinical observations, some commonly used herbs have been demonstrated to interact with Western therapy.[63] Herbal medicines can cause abnormal test results and confusion in proper diagnosis. Herbal medicines can alter test results by direct interference with certain immunoassays. Drug-herb interactions can result in unexpected concentrations of therapeutic drugs.[64,65] The frequency of interactions must not be underestimated. Yoon and Schaffer[66] identified drug-drug interactions (DDIs) using a Web-based pharmaceutical program. At least one moderate or high-risk DDI was identified in 74% of participants, with 136 total DDIs identified. Fifty-two percent (71) of total DDIs were between prescribed and OTC or herbal products with 63% (45) of these involving nonsteroidal antiinflammatory drugs.

Bush et al.[67] performed a review of the patient's chart for evidence of an observed adverse herb-drug interaction. Out of 804 patients surveyed, 122 (15%) used herbal medicines. Eighty-five potential adverse herb-drug interactions were found in 49 patients (40% of herbal medicine users). Twelve possible adverse herb-drug interactions in 8 patients (7% of herbal medicine users) were observed. In all 12 cases, the severity scores were rated as mild, including 8 cases of hypoglycemia in diabetics taking nopal (prickly pear cactus).

Using a matrix of 165 possible drug-herb interaction pairs (15 therapeutic drug classes by 11 herbal products), Brazier and Levine[68] identified 51 (31%) interactions. Twenty-two of these 51 drug-herb pairs (43%) were supported by randomized clinical trials, case-control studies, cohort studies, case series, or case studies. The remaining interaction pairs reflected theoretic reasoning in the absence of clinical data. Most interactions were pharmacokinetic, most of which affected cytochrome p450 enzymes.

While cytochrome P450-mediated herb-drug interactions have been extensively characterized, the effects of herbal extracts and constituents on UDP-glucuronosyl transferase (UGT) enzymes have been less studied. Mohamed and Frye[69] evaluated current evidence on the glucuronidation of phytochemicals and the potential for UGT-mediated herb-drug interactions with top-selling herbal supplements in U.S.A. and Europe. *In vitro* and animal studies indicate that cranberry, *Ginkgo biloba*, grape seed, green tea, hawthorn, milk thistle, noni, soy, St. John's wort, and valerian are rich in phytochemicals that modulate UGT enzymes. However, the *in vivo* consequences of these interactions are not well understood as yet.

## 3 Perspectives for a 'Take Actions' Plan

### Development of Educational Programs and Databases

Self-administration of medication suggests that individuals are functionally and cognitively competent to manage their health care. However, this is not always the case. Therefore, physicians, pharmacists, nurses, midwifes, and other health care professionals have a critical responsibility assessing the self-care ability of their patients.[70,71] Databases are available for pharmacists with information on action, side effects and toxicities as well as herb-drug interactions. A well known database is the DMDI-ABDA database of the German Institute for Medical Documentation and Information (http://www.dimdi.de/static/de/amg/abda/index.htm). Some health care professionals working with patients, may find themselves confronted with questions about herbal remedies, yet may feel unable to answer them or be unsure where to find information. However, with the generally increased use of complementary therapies, efforts are necessary to facilitate health professionals to offer accurate, comprehensive and evidence-based information to patients.[72] Physicians should routinely ask patients about their use of dietary supplements when starting or stopping a prescription drug, or if unexpected reactions occur. Patient and physician education, and regular medication monitoring are essential to prevent polypharmacy. Since there are still scant guidelines about the assessment of safe self-administration of conventional and herbal medication, novel initiatives for improvement are required.[73]

### Guidelines and Phytovigilance

There is a need for established guidelines regarding discontinuation of selected natural products prior to surgery and further education is needed concerning the perioperative implications of natural products.[20] Good communication with surgical patients, including the administration of a presurgical questionnaire to help identify any use of herbal medications, is very important.

The absence of precise prescribing guidelines, and the risk of self-prescribed medication justify the introduction of **'phyto-vigilance'**. Self-medication-induced morbidity might be prevented by the documentation of medicines in the medical record and review of the medical record before new medications are prescribed. The relevance of this topic is illustrated in a survey of Jaski et al.. [74] Documentation and review by primary care physicians of patients using prescription drugs, OTC products, nutritional supplements, and herbal treatments were surveyed among 1802 internists and family practitioners from the American Medical Association Physician Masterfile. Almost all of the 655 responders reported reviewing prescription medications before prescribing a new therapy (99.8%), but only 86% reported reviewing OTCs at the same time. Fewer than half of physicians reported reviewing nutritional supplements or herbal treatments before prescribing a new therapy. Since the response rate of physicians was low, the authors concluded that their results probably overestimated actual rates of documentation and review. Review and documentation of nonprescription substances are uncommon in primary care practice.

Adverse drug interactions are among the leading causes of death in hospitalized patients. In children and older adults undetected food/herb-drug interactions may lead to serious morbidity and mortality and be misdiagnosed as chronic disease progression.[75] Recent recognition of the effects of certain foods on many drugs metabolized by CYP450 families or drugs susceptible to chelation and adsorption have increased awareness for prevention of food-drug interactions. Self-medications with non-prescription herbal remedies, nutritional supplements, and genetic polymorphisms in drug metabolism enzymes increase the need to consider food/herb-drug interactions. Prevention of adverse events from herb-drug interactions requires clinical monitoring in high-risk regimens and populations. The nutritional status has an important impact on the quality of life as well as appropriate responses to drug therapy. Both diet-drug histories and counseling are needed.[75]

Faced with the extensive distribution of many herbal preparations and the risk of self-medication, consumers, clinicians, pharmacists, and health care professionals should be vigilant with potentially toxic herbal OTC products. Personal use of dietary supplements and herbal OTC products correlated with a twofold increase in the likelihood that a pharmacist would recommend a dietary supplement to a patient.[76] This indicates that there is a need among pharmacists for dietary supplements information to be included in pharmacy computer systems, specifically to check for interactions against the patient's drug profile. Although practitioners as well as journal articles are preferred information sources, the internet and the personal talk with the pharmacist are also important sources. Pharmacists should provide competent information of non-prescription herbal products and dietary supplements. It is essential for pharmacists to counsel patients and the general public on the benefits and risks associated with supplement use.[77]

### Information Centers

Information centers for consumers and general practitioners shall meet the strong demand for counseling and providing high-quality, evidence-based information on exposures of risk groups. An illustrative example for such an information center is “MotherSafe”, which was established at the Royal Hospital for Women (Randwick NSW, Australia).[78] MotherSafe is an information service on teratogenic exposures during pregnancy and lactation.

To obtain a rational basis about the needs for such information centers, the interests of custumors have to be known. A retrospective review of 1087 queries submitted online to an “Ask your Pharmacist” (AYP) service has been published by Assemi et al..[79] The authors described consumer demographics regarding gender, age, patient relationship to consumer, allergies, medical conditions, products, and evaluated the types of submitted questions. Consumers of all ages used the AYP services for both acute and chronic conditions. Most consumers asked questions related to their own health or medications. Consumers were more likely to ask questions related to prescription and OTC medications than to botanical and dietary supplements. Consumers were primarily interested in drug efficacy and adverse effects.

### Clinical Trials

Many data available on the effects of natural medicines is only anecdotal. Numerous case reports and information on compassionate use of herbal products might indicate that herbal products are effective, but current quality control standards are highly variable. In order to ensure quality control and standardization of products, preparations manufactured by companies that adhere to good practice standards (GSP, GAP, GMP, etc.) are advisable.

There is a lack of controlled clinical trials and the incidence of adverse effects is not clarified. Many plant products do not seem to lead to toxic effects in everyone taking them, and they commonly lack a strict dose-dependency.[56] Herbal products should fulfill the criteria of evidence-based medicine to gain full recognition in Western medicine. Therefore, placebo-controlled, double blind and randomized clinical trials are needed to provide convincing evidence on safety and efficacy of herbal products. Current clinical trial protocols frequently do not reflect the "typical" general elderly population are needed.[14]

**Open Access** This article is distributed under the terms of the Creative Commons Attribution License which permits any use, distribution, and reproduction in any medium, provided the original author(s) and source are credited.
